# Vaccine-Associated Measles Encephalitis in Immunocompromised Child, California, USA

**DOI:** 10.3201/eid2804.212357

**Published:** 2022-04

**Authors:** Cristina Costales, Malaya K. Sahoo, ChunHong Huang, Carolina V. Guimaraes, Donald Born, Lauren Kushner, Hayley A. Gans, Thuy A. Doan, Benjamin A. Pinsky

**Affiliations:** Stanford University School of Medicine, Stanford, California, USA (C. Costales, M.K. Sahoo, C. Huang, C.V. Guimaraes, D. Born, L. Kushner, H.A. Gans, B.A. Pinsky);; Proctor Foundation, San Francisco, California, USA (T.A. Doan)

**Keywords:** measles, viruses, vaccine-preventable diseases, meningitis/encephalitis, vaccination, stem cell transplant, immunocompromised patient, California, vaccines, United States

## Abstract

We report a fatal case of vaccine-associated measles encephalitis in an immunocompromised child in California, USA. The infection was confirmed by whole-genome RNA sequencing of measles virus from brain tissue. We observed biased matrix-gene hypermutation consistent with persistent measles virus central nervous system infection.

Measles is a highly contagious, vaccine-preventable, systemic viral disease caused by measles virus (MV), an enveloped, single-stranded, negative-sense RNA virus in the genus *Morbillivirus*, family Paramyxoviridae. MV may cause persistent central nervous system (CNS) infections that result in fatal neurologic diseases, such as subacute sclerosing panencephalitis and measles inclusion body encephalitis. Live-attenuated MV-containing vaccines, such as measles-mumps-rubella (MMR), are administered in a 2-dose series and are estimated to be >95% effective in preventing clinical measles (*1*,*2*). Serious adverse events are relatively uncommon after MV vaccination; rare reports have documented measles-like illness, predominantly in immunocompromised children (*2*–*5*). To our knowledge, before the case we report, only 1 sequence-confirmed, postvaccination MV CNS infection had been reported (*6*). 

A previously healthy infant received dose 1 of the MMR ProQuad vaccine (Merck, https://www.merck.com) at her 1-year well-child visit. Over the following week, the patient experienced fevers, and acute myeloid leukemia was diagnosed. During induction chemotherapy, a diffuse morbilliform rash developed. A nasopharyngeal swab sample was positive for MV RNA by a laboratory-developed multiplex quantitative reverse transcription PCR (*7*). We detected all 3 genomic targets: the nucleoprotein, hemagglutinin, and large protein genes. In addition, the carboxyl-terminal nucleoprotein typing sequence was identical to the MV component of the ProQuad vaccine, the Edmonston-Enders (Moraten) strain (*8*). The patient received intravenous immunoglobulin and vitamin A. The rash resolved after ≈8 days. 

Four months after her initial acute myeloid leukemia diagnosis, the patient received a paternal haploidentical stem cell transplant. One month after the transplant, she experienced altered mental status; magnetic resonance imaging showed abnormal signals, and positron emission tomography showed hyperperfusion involving the insula and thalamus (Appendix Figure 1). The patient experienced respiratory decompensation of suspected neurologic origin associated with brain lesion progression involving the right mesial temporal lobe, which was biopsied. We cut 5-μm sections of formalin-fixed, paraffin-embedded (FFPE) brain tissue and mounted them on glass slides. We prepared hematoxylin and eosin–stained sections and glial fibrillary acidic protein immunoperoxidase-stained sections (GFAP Clone GA5; Leica Biosystems, https://www.leicabiosystems.com) by standard methods for diagnostic neuropathology evaluation. Histology showed deep gray and white matter with astrogliosis (Appendix Figure 2) but without notable inflammation, neoplastic cells, or definite inclusions. 

To evaluate for CNS MV, we extracted total nucleic acids from FFPE brain tissue scrolls using a Quick-RNA FFPE kit (Zymo Research, https://www.zymoresearch.com) according to manufacturer instructions. MV quantitative reverse transcription PCR detected all 3 genomic targets. We then performed metagenomic RNA sequencing as described elsewhere (*9*). In brief, we converted 5 μL of extracted total nucleic acids to complementary DNA and prepared sequencing libraries using the NEBNext RNA ULTRA II kit (New England Biolabs, https://www.neb.com) according to manufacturer instructions. We sequenced samples on the NovaSeq 6000 system (Illumina, https://www.illumina.com) using 150-nt paired-end sequencing and aligned them to the MV vaccine complete genome (GenBank accession on. FJ211583) using the bwasw module in Burrows-Wheeler Aligner version 0.7.9a-r786 (http://bio-bwa.sourceforge.net) with match score 2 and mismatch penalty −3 (options -a2 -b3). We used a custom python script to call mutations from the sequence alignment file. We discarded mutations <5% as noise. The consensus whole-genome sequence (Genbank OL473966) was generated from the sequence alignment file using SAMtools version 0.1.19 mpileup (http://www.htslib.org/doc/samtools-mpileup.html), followed by bcftools vcf2fq (https://github.com) using default parameters. 

Metagenomic RNA sequencing of brain tissue acquired 50.7 million reads, 190,303 of which aligned to Edmonston-Enders (Moraten) MV. We obtained 500× coverage over 98.2% of the whole MV genome with mean coverage of 1,540× reads. We identified no known hyperfusogenic fusion gene mutations (*10*). However, 38.0% (68/179) of uracil-containing codons in the matrix (M) gene contained >1 uracil-to-cytosine mutation at levels ≥5% (Figure). Despite treatment with intravenous ribavirin, intrathecal interferon-α, and inosine pranobex, the patient experienced persistent dysautonomia, respiratory failure, and myoclonus; without conceivable neurologic recovery, she was extubated and died shortly thereafter. The family declined autopsy. 

**Figure:**
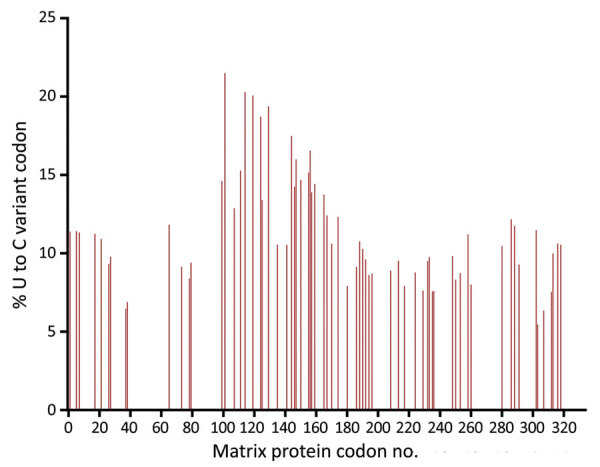
Sequenced vaccine-strain measles virus matrix protein gene from a 1-year-old patient’s brain tissue, California, USA. Results show biased hypermutation of uracil-containing codons, with >1 U-to-C mutation at levels ≥5%, consistent with hypermutation of the matrix gene as found in central nervous system measles virus infections such as subacute sclerosing panencephalitis and measles inclusion body encephalitis. C, cytosine; U, uracil.

Unique to this case was identification of biased M-gene hypermutation. In the previously reported case of sequence-confirmed, postvaccination MV CNS infection, M-gene hypermutation was not observed, likely because of limitations of Sanger sequencing for detecting minority variants (*6*). However, biased M-gene hypermutation is a characteristic of prolonged CNS replication in subacute sclerosing panencephalitis and measles inclusion body encephalitis. Because neurons lack SLAM/CD150 (signaling lymphocytic activation molecule/cluster of differentiation 150) and nectin4, the known receptors for wild-type MV, reduced M-gene expression may contribute to CNS persistence and disease progression by MV cell-to-cell transmission (*10*). The biased M-gene hypermutation we observed in this fatal case of postvaccine MV encephalitis confirmed viral CNS persistence. However, because Edmonston vaccine strains also use the ubiquitous CD46 receptor, the role of these genomic changes in neuropathogenesis remains to be determined. 

The concurrence of live-attenuated MV vaccination and impending diagnosis of hematologic malignancy in this case was an unfortunate but uncommon circumstance. Nevertheless, clinicians should be vigilant for signs and symptoms of immunocompromise before administering live-attenuated vaccines. 

Appendix Additional information about vaccine-associated measles encephalitis in immunocompromised child, California, USA.
