# Supplementary material for: Vaccine-Associated Measles Encephalitis in Immunocompromised Child, California, USA
Source: Emerg Infect Dis. 2022 Apr;28(4):906–8. doi: 10.3201/eid2804.212357 (PMC8962891; doi:10.3201/eid2804.212357)

Article DOI: https://doi.org/10.3201/eid2804.212357

Vaccine-Associated Measles Encephalitis in Immunocompromised Child, California, USA

Appendix

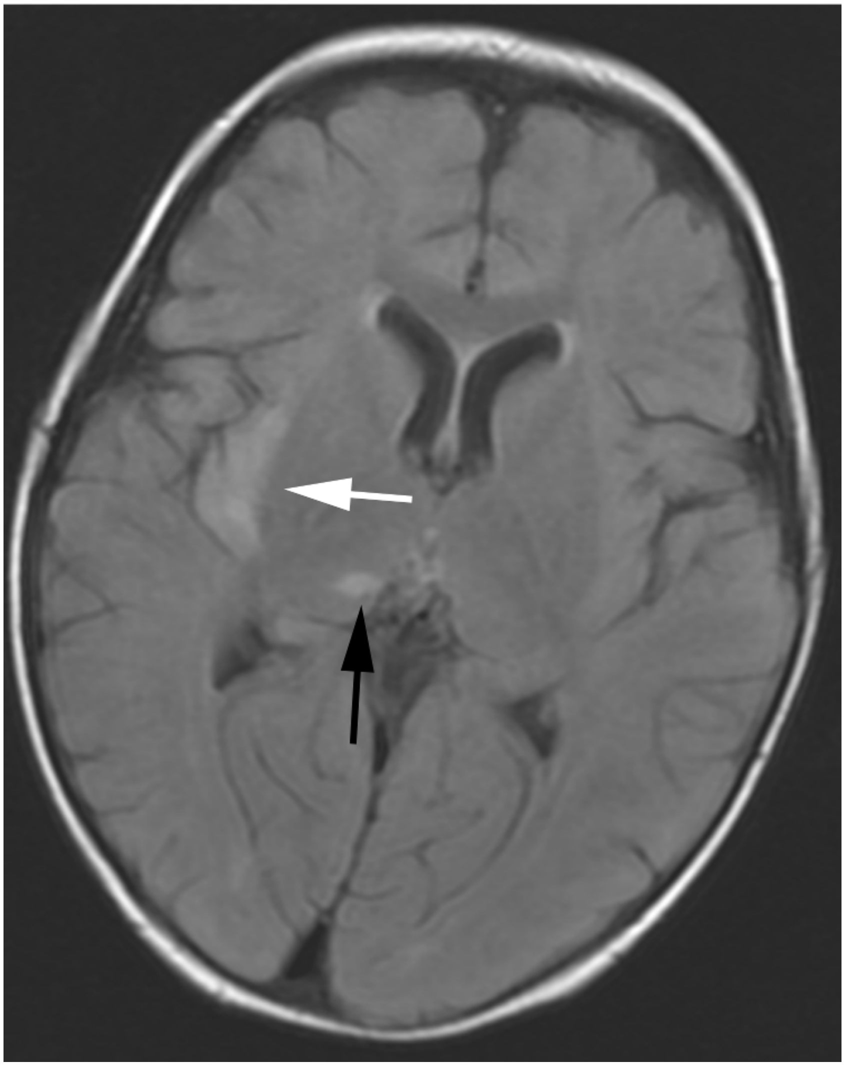

Appendix Figure 1. Axial fluid-attenuated inversion recovery (FLAIR) image at the level of the thalamus showing multifocal hyperintensities in the right insular subcortical white matter and cortex (white arrow) and right thalamus (black arrow).

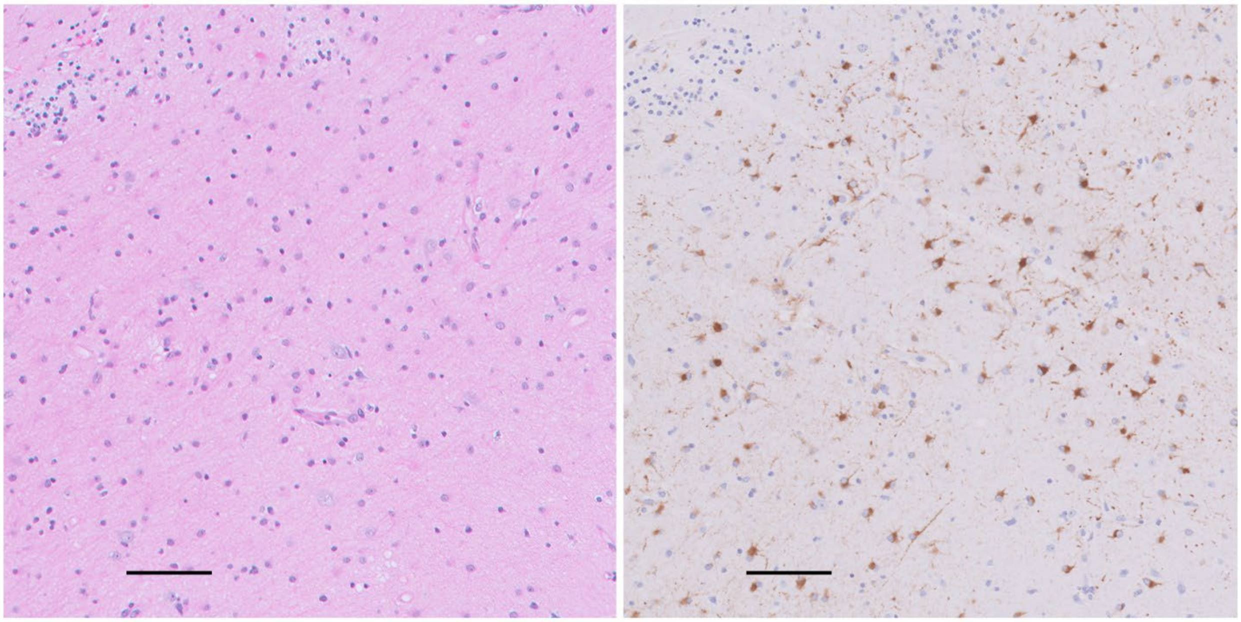

Supplement: Appendix — Additional information about vaccine-associated measles encephalitis in immunocompromised child, California, USA. [file 21-2357-Techapp-s1.pdf]
